# Quantitative Muscle MRI as an Assessment Tool for Monitoring Disease Progression in LGMD2I: A Multicentre Longitudinal Study

**DOI:** 10.1371/journal.pone.0070993

**Published:** 2013-08-14

**Authors:** Tracey A. Willis, Kieren G. Hollingsworth, Anna Coombs, Marie-Louise Sveen, Søren Andersen, Tanya Stojkovic, Michelle Eagle, Anna Mayhew, Paulo L. de Sousa, Liz Dewar, Jasper M. Morrow, Christopher D. J. Sinclair, John S. Thornton, Kate Bushby, Hanns Lochmüller, Michael G. Hanna, Jean-Yves Hogrel, Pierre G. Carlier, John Vissing, Volker Straub

**Affiliations:** 1 Institute of Genetic Medicine, Newcastle University, Newcastle upon Tyne, United Kingdom; 2 Institute of Cellular Medicine, Newcastle University, Newcastle upon Tyne, United Kingdom; 3 Department of Neurology, University of Copenhagen, Copenhagen, Denmark; 4 AP-HP, Institute of Myology, Groupe Hospitalier Pitié-Salpêtrière, Paris, France; 5 NMR Laboratory, Institute of Myology and CEA, Paris, France; 6 MRC Centre for Neuromuscular Diseases, Department of Molecular Neurosciences, UCL Institute of Neurology, London, United Kingdom; 7 Institute of Myology, Groupe Hospitalier Pitié-Salpêtrière, Paris, France; The Hospital for Sick Children, Canada

## Abstract

**Background:**

Outcome measures for clinical trials in neuromuscular diseases are typically based on physical assessments which are dependent on patient effort, combine the effort of different muscle groups, and may not be sensitive to progression over short trial periods in slow-progressing diseases. We hypothesised that quantitative fat imaging by MRI (Dixon technique) could provide more discriminating quantitative, patient-independent measurements of the progress of muscle fat replacement within individual muscle groups.

**Objective:**

To determine whether quantitative fat imaging could measure disease progression in a cohort of limb-girdle muscular dystrophy 2I (LGMD2I) patients over a 12 month period.

**Methods:**

32 adult patients (17 male;15 female) from 4 European tertiary referral centres with the homozygous c.826C>A mutation in the fukutin-related protein gene (*FKRP*) completed baseline and follow up measurements 12 months later. Quantitative fat imaging was performed and muscle fat fraction change was compared with (i) muscle strength and function assessed using standardized physical tests and (ii) standard T1-weighted MRI graded on a 6 point scale.

**Results:**

There was a significant increase in muscle fat fraction in 9 of the 14 muscles analyzed using the quantitative MRI technique from baseline to 12 months follow up. Changes were not seen in the conventional longitudinal physical assessments or in qualitative scoring of the T_1_w images.

**Conclusions:**

Quantitative muscle MRI, using the Dixon technique, could be used as an important longitudinal outcome measure to assess muscle pathology and monitor therapeutic efficacy in patients with LGMD2I.

## Introduction

Limb-girdle muscular dystrophy 2I (LGMD2I) is an autosomal recessive disease frequently caused by a homozygous founder mutation (c.826C>A) in the fukutin-related protein gene (*FKRP*) [1], [2]. LGMD2I is one of the most common forms of LGMD in northern Europe [3]. The disease is heterogeneous with age of onset, rate of progression and degree of severity varying greatly. Respiratory and cardiac complications are common, and can occur independently from the skeletal muscle weakness [4], [5], [6], [7]. As putative therapies for muscular dystrophies continue to be developed, identification of objective and sensitive markers of longitudinal change is important.

Presently, measures based on physical function testing (such as the 6 minute walk distance, time to rise from a chair, muscle force measurements or composite scores) are used as primary end-points in therapy trials. However, these measurements are dependent on patient motivation, may be subject to learning effects and have limited reproducibility, reducing their power to detect small differences in clinical trials. These functional measures also typically combine the efforts of several different muscle groups, whereas it is known that muscle pathology can advance at markedly different rates in different muscles.

Previous magnetic resonance imaging (MRI) research has concentrated on small cross-sectional studies employing standard T1-weighted imaging interpreted by radiological scoring and reporting the patterns of muscle involvement in specific muscular dystrophies [8], [9], [10], [11], [12]. However, qualitative scoring is subjective, typically limited to a scale of 4–6 grades, and hence masks small changes in muscle pathology. The quantitative Dixon technique allows fat fraction analysis [13], correcting for inhomogeneities in magnetic and radiofrequency fields which make signals on standard T_1_w images difficult to quantify (see Supporting Information in File S1 for more detail). Such measurements are independent of patient motivation and are highly reproducible.

In this study both qualitative T_1_w imaging and quantitative fat measurement, using the Dixon technique, together with functional muscle assessment and strength tests, were acquired at baseline and 12 months follow-up to detect the small pathological changes expected. We present the results of the first longitudinal 12-month natural history study of muscle fat content in a large multinational cohort of LGMD2I patients. Muscle pathology progression over 12 months can be detected by quantitative MRI but not by assessing muscle strength or function.

## Materials and Methods

### Setting and Study Population

This study complied with the Declaration of Helsinki, was approved by local ethics committees and written informed consent was obtained from each participant at the 4 centers: ethical approval for the Newcastle upon Tyne and London centers was provided by the Newcastle and North Tyneside Research Ethics Committee 2 (reference 09/H0907/29); the Regional Committee on Biomedical Ethics of Copenhagen (reference H-B-2009-061) provided approval for the Copenhagen centre; and the Local Ethics Committee CPP-Ile-de-France VI (reference 2009-A00808-49) approved the Paris center. Thirty eight ambulant adults with a genetically confirmed diagnosis of LGMD2I (19M:19F age 18–64 years, mean ages of 40.9 years and 40.0 years respectively and disease duration 0–49 years) were recruited. Thirty two patients (17M:15F age 19–65 years, mean ages of 40.3 years and 41.9 years) completed the follow up assessment after 12 months including the MRI and the full standardized physical and functional assessment: see Supporting Information in File S1 for details of loss to follow-up. Inclusion criteria for the patients were: identical genetic diagnosis with homozygous c.826C>A *FKRP* mutations, ambulant for more than 50 metres, no ventilator requirements and the ability to lie flat with no contraindications for MRI scanning. Data were acquired locally at the four centers between June 2009 and April 2011, using pre-agreed standardized MRI and clinical and functional assessments with a standardized reporting method.

A physiotherapy assessment was performed in all patients at the time of the MRI. Strength testing of the lower limb on the dominant side using a hand-held myometer (MicroFET) [14] included knee extension, knee flexion, hip abduction and hip adduction. Timed tests included a 10 metre walk/run [15], Timed Up and Go test (TUG) [16] and a six minute walk distance (6MWD) [17], [18]. Forced vital capacity (FVC) was measured as the best of three attempts using a hand-held spirometer, both sitting and supine, and expressed as percentage of the expected value based on height. Protocols and training manuals were produced and teleconferences organised to ensure uniformity of approach.

### MRI Acquisition

The acquisition protocol comprised (i) standard T_1_w imaging allowing whole muscle qualitative evaluation and (ii) quantitative fat imaging by the Dixon technique optimized for muscle fat-fraction analysis: see Supporting Information in File S1 for further detail on the method and advantages of Dixon imaging. Acquisitions were performed at 3.0 T (Philips Achieva, Siemens TIM Trio) with local surface-array receive coils for signal detection. The use of different MRI scanners from different vendors across the 4 participating sites necessitated detailed pre-trial quality assurance activity using both water/fat phantoms and healthy subjects to ensure quantitatively equivalent MRI results across the centers (see File S1).

#### (i) T_1_w imaging

Axial T_1_w imaging was performed with a turbo spin echo sequence (repetition time (TR)/echo time (TE) = 671/10 ms, (Newcastle/Paris) or 16 ms (London), 12 ms (Copenhagen), number of averages 2, acceleration factor 3, slice thickness 5 mm, interslice gap 10 mm, 256×192 matrix interpolated to 512×384). FOV 410 mm using multiple stacks to cover both legs from the ankle to the pelvic crest.

#### (ii) Quantitative Dixon imaging

Spoiled gradient-echo sequences were used in the axial plane. Protocol details varied slightly between sites: Newcastle/London: 3 point Dixon images acquired in 2D with TR/TE = 100/3.45, 4.6, 5.75 ms, flip angle = 10 degrees, 10 slices of 10 mm slice thickness, 5 mm gap; Paris: 3D, 64 slices of 5 mm slice thickness with TR/TE = 10/2.75,3.95,5.15 ms, flip angle = 3 degrees; Copenhagen: as per Paris, but with 36 slices per 3D acquisition, 2-point Dixon with correction for B_0_ inhomogeneity [19]. Optimal TEs were determined locally due to differences in the actual precise B_o_ magnitude of Siemens and Philips scanners. Images were collected at mid-lower leg and mid-thigh: the central plane of acquisition was defined with respect to bone landmarks as follows: legs were positioned with the patella anterior; the lower leg images were centered by finding the broadest part of the lower leg muscle, and recording the distance from the lower border of patella; thigh images were centered by locating superior border of patella, and the anterior superior iliac spine, with centering 1/3 of the distance superior to the patella; the distances were recorded for prescription of follow-up scans. Each leg was imaged individually using a 160×160 matrix interpolated to 256×256, FOV 200×200 mm: in Paris, it was possible to scan both legs together at the same resolution using FOV 448×244 mm. The data was analyzed off-line to produce separate fat and water images [19], [20] from which quantitative fat-fraction maps were produced by expressing the fat signal as a percentage of the total signal per voxel.

#### (iii) Interpretation

The images were analyzed by a consultant neurologist (TW). The T_1_w images were assessed on an individual muscle basis and graded according to the scale published by Mercuri et al. [21], [22] (Table 1), assessing 14 muscles in the leg (9 in the thigh and 5 in the lower leg) and the gluteus maximus muscle. The quantitative fat-fraction maps were analyzed by defining regions of interest (ROIs) encompassing the full cross-section of individual muscles on the separated water image at the midpoint of both the lower leg and thigh for both baseline and follow-up datasets in 14 muscles, avoiding partial volume effects at the boundaries. To assess the inter-observer variability and hence objectivity of the measurements made, ROI assessment of all muscles in the quantitative imaging and the scoring of qualitative grades in a subset of 8 patients was also performed by KGH, an MR physicist with 6 years’ experience of muscle imaging. Bland-Altman analysis was performed to assess the objectivity of the method for the quantitative images [23]. The inter-observer Bland-Altman analysis gave an inter-observer bias of 0.04% (no systematic bias) and an inter-observer repeatability coefficient of 1.43% (95% of comparisons will fall within this range). For the qualitative scoring, the two observers were in agreement for 65% of muscle groups scored. Agreement was highest at the extremes of the grades and least in the middle, where only 39% agreement was achieved in scoring grade 2b.

**Table 1:** Description of the qualitative muscle grading scale (21).

Grade	Description
**0**	Normal appearance
**1**	Early moth-eaten appearance with scattered small areas of increased signal
**2a**	Late moth-eaten appearance with numerous discrete areas of increased signal with beginning confluence, comprising less than 30% of the volume of the individual muscle
**2b**	Late moth-eaten appearance with numerous discrete areas of increased signal with beginning confluence, comprising 30–60% of the volume of the individual muscle
**3**	Washed-out appearance, fuzzy appearance due to confluent areas of increased signal
**4**	End stage appearance, muscle replaced by increased density of connective tissue and fat, with only a rim of fascia and neurovascular tissue distinguishable.

### Statistical Methods

The Shapiro-Wilk test demonstrated that the fat fraction measurements were not normally distributed. The average fat fractions in the group for a given muscle were therefore characterised by the median, with minimum and maximum values quoted. Non-parametric statistical tests were used for analysis using the SPSS version 17.0 software (IBM, USA). Comparison of fat-fraction changes between paired baseline and follow up scans were tested using the Wilcoxon signed-rank test. Comparison of the functional changes between baseline and follow up were tested using the Wilcoxon signed-rank test. Statistical significance was taken to be p<0.05.

## Results

### Analysis of T_1_w Images by Grading

Pelvis, thigh and lower leg T_1_w images were analyzed both at baseline and at 12 months in the 32 patients for which MRI was available at both time points. The biceps femoris short head (BFSH) in 3 individuals could not be graded due to poor visualisation of the muscle. Table 2 highlights that there is no significant difference between the grades at baseline and follow-up by scoring the muscles qualitatively. The majority of the muscles remained the same grade at both baseline and follow up. Table S1 in File S1 shows the percentage of patients scoring grades 0–4 for all 15 muscles on T_1_w MRI and the median grade scored in the 32 patients at follow-up.

**Table 2:** Median qualitative grades for selected muscles at baseline and at 12 months.

Muscle	Baseline median grade	12 month median grade	Number with same/increased/decreased grade	P value
GM	4	4	25/4/3	.705
BFLH	4	4	29/1/2	.564
ST	3	4	23/4/5	.739
SM	3	3	20/5/7	.509
BFSH*	2b	2b	20/4/5	.739
SAR	2b	2b	19/8/5	.405
VM	2b	3	16/8/8	.819
GRAC	2a	2b	23/4/5	.739
VL	2b	2b	17/10/5	.346
RF	2a	2b	22/6/4	.527
MG	2b	2b	21/7/4	.218
LG	2b	2b	18/10/4	.073
PL	2b	2a	15/7/10	.655
SOL	2a	2b	23/5/4	.739
TA	2a	2a	26/3/3	.739

Number of patients with the same, an increased or decreased qualitative grade at the 12 months follow up compared to the baseline grade is given. Non-parametric paired Wilcoxon signed rank test was used to assess change.

* BFSH was graded in 29 participants. BFSH in 3 subjects could not be graded due to poor visualisation. GM = Gluteus Maximus, BFLH = Biceps Femoris long head, ST = Semitendinosus, SM = Semimembranosus, BFSH = Biceps Femoris short head, SAR = Sartorius, VM = Vastus Medialis, GRAC = Gracilis, VL = Vastus Lateralis, RF = Rectus Femoris, MG = Medial Gastrocnemius, LG = Lateral Gastrocnemius, PL = Peroneus Longus, SOL = Soleus, TA = Tibialis Anterior.

For the gluteus maximus muscle 81% of the patients scored grade 3 or 4 at baseline and follow up. Even in mildly affected patients, the gluteus maximus muscle was often severely affected early in the disease and did not appear to change longitudinally.

### Quantitative Analysis – Dixon Technique

**Figure 1:**
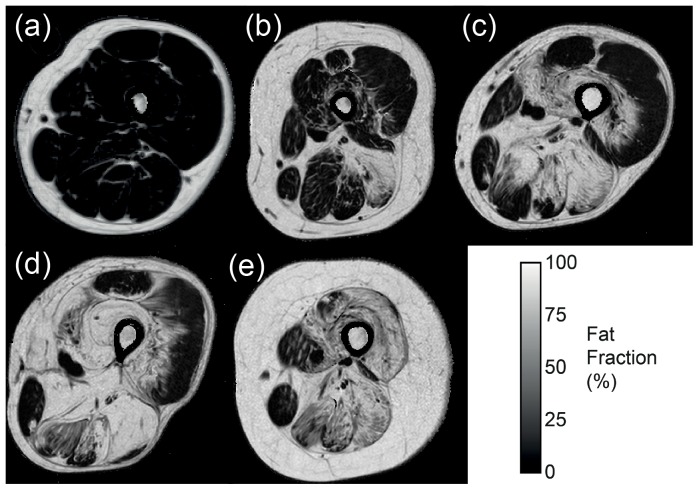
Quantitative fat fraction images of the thigh in LGMD2I patients showing the wide range of severity in fat replacement at baseline, from minimal involvement (a) to sparing of only gracilis and sartorius (e).

A total of 14 muscles per patient, nine muscles at mid thigh level and five muscles at mid lower leg level, were analysed in all 32 patients at baseline and follow-up (Table 3). Gluteus maximus muscle data was not acquired with the Dixon technique. The distribution of muscle pathology was broadly similar at baseline and follow-up and showed a broad spectrum of severity (Figure 1): some muscles were more severely affected such as the biceps femoris long head muscle (median fat fraction 71.6% at baseline), whilst others demonstrated mild involvement, as in the tibialis anterior muscle (median fat fraction 5.5% at baseline).

**Table 3:** Median values of the fat fractions at baseline and follow up.

Muscle	Baseline min	Baseline max	Baseline median	12 month min	12 month max	12 monthmedian	Paired diff. (sig)
**BFLH** §	1.5	97.3	71.6	2.2	94.4	75.2	**0.004**
**ST** §	2.3	100	55.7	2.1	96.1	59.7	**0.021**
**SM** §	2.6	94.1	49	2.9	95.6	54.2	**0.015**
**BFSH**	2.7	78.1	25.5	4.1	82.3	24.9	0.06
**SAR** § *	0.85	88.9	24.2	3.4	87.5	25.3	**0.010**
**VM**	1.1	89.1	25.6	0.8	83.5	30.9	0.065
**GRAC** § *	2.3	81.7	25.3	3.9	84.2	26.6	**0.018**
**VL** § *	0.6	82.1	15.6	1.2	82.1	20.9	**0.025**
**RF** § *	0.4	81.3	10.9	0.8	82.1	12.3	**0.028**
**MG** § *	1.1	90.3	21.7	1.3	90.3	22.6	**0.009**
**LG** §	0.8	88.4	19.3	0.8	87.8	23.9	**0.009**
**PL**	2.8	55	15.1	3.2	61.8	14	0.896
**SOL**	1.5	84.9	9.1	2.1	86	10.9	0.246
**TA**	1.4	24.6	5.5	1.3	23.5	5.2	0.627

The non-parametric paired Wilcoxon signed rank test was used to test significance.

§ significant differences between baseline and 12 month follow-up.

* possible muscles for future longitudinal analysis; In lower leg; MG, In thigh; SAR, GRAC, VL and RF.

BFLH = Biceps Femoris long head, ST = Semitendinosus, SM = Semimembranosus, BFSH = Biceps Femoris short head, SAR = Sartorius, VM = Vastus Medialis, GRAC = Gracilis, VL = Vastus Lateralis, RF = Rectus Femoris, MG = Medial Gastrocnemius, LG = Lateral Gastrocnemius, PL = Peroneus Longus, SOL = Soleus, TA = Tibialis Anterior.

**Figure 2:**
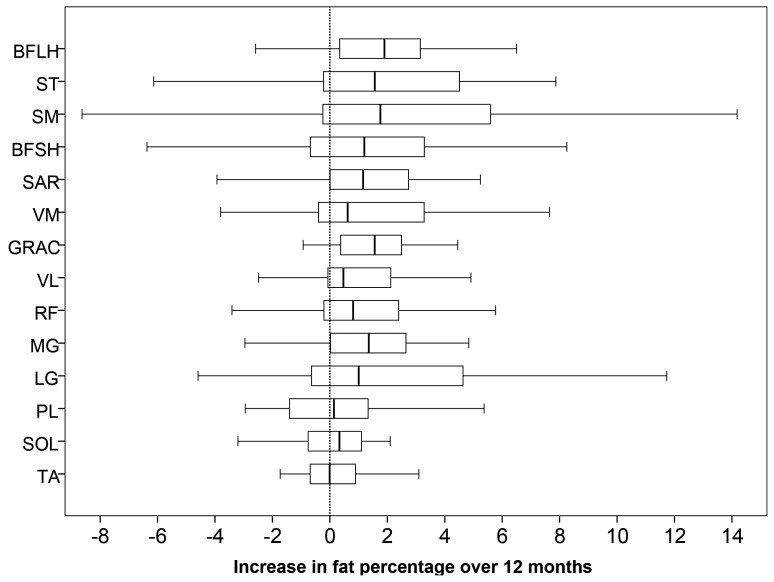
Quantitative fat fraction differences from baseline to 12 month follow-up. The box indicates the lower and upper quartiles, with the median change with time represented as the bar within the box. The bars enclose the outliers.

**Figure 3:**
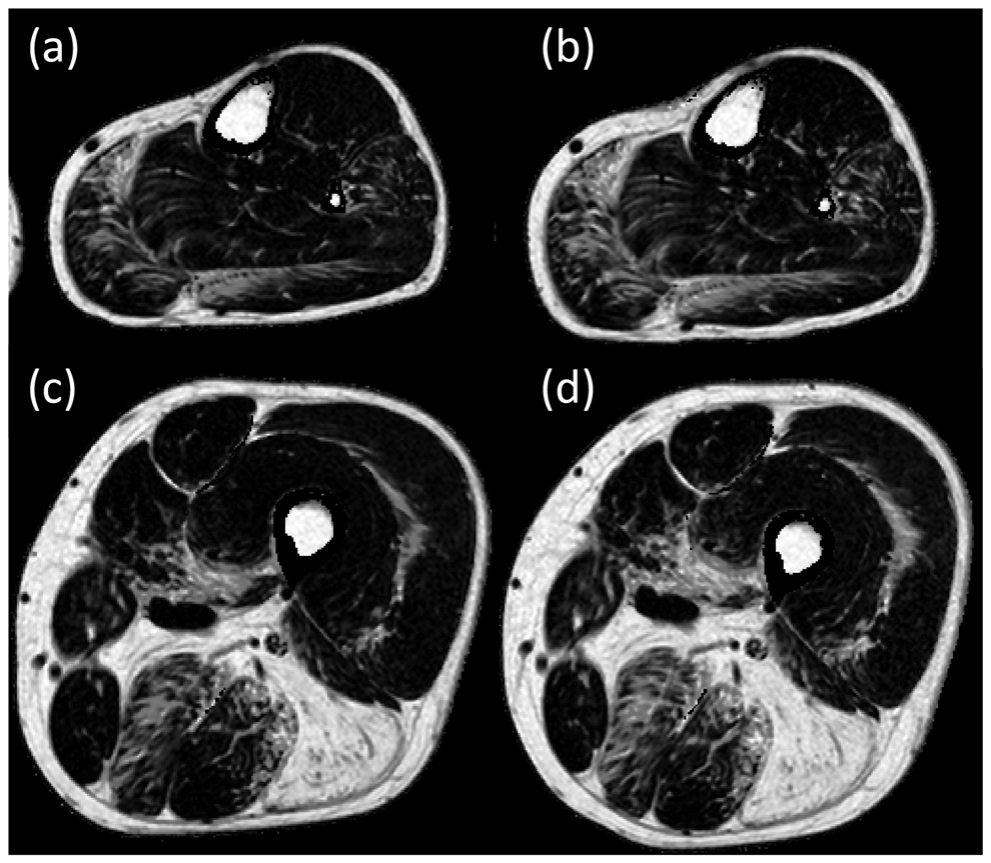
Quantitative fat fraction images at baseline in an individual patient (a – mid lower leg level, c – mid thigh level) and at 12 months follow-up (b – mid lower leg level, d – mid thigh level). Analysis reveals an increase in fat fraction of the medial and lateral gastrocnemius, peroneus longus, vastus lateralis and medialis, semimembranosus, semitendinosus, sartorius and gracilis.

**Figure 4:**
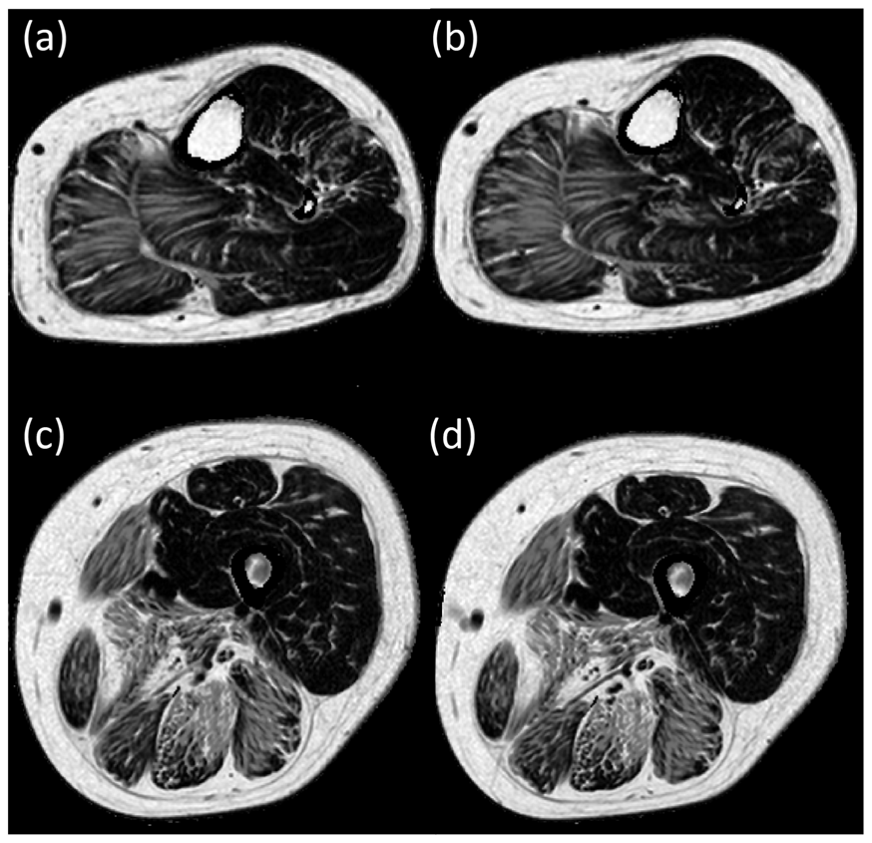
Quantitative fat fraction images at baseline in an individual patient (a – mid lower leg level, c – mid thigh level) and at 12 months follow-up (b – mid lower leg level, d – mid thigh level). Analysis reveals an increase in fat fraction of the lateral and medial gastrocnemius, sartorius, gracilis, semitendinosus, rectus femoris and vastus lateralis.

The fat-fractions in 9 out of the 14 muscles increased significantly from baseline to follow-up, these being the medial and lateral gastrocnemius muscle in the lower leg, and the sartorius, gracilis, semimembranosus, semitendinosus, vastus lateralis, rectus femoris and biceps femoris long head muscle in the thigh. Changes in the fat fraction in other muscles did not reach statistical significance over 12 months. Figure 2 illustrates the difference in the fat fractions between baseline and follow-up for each individual muscle, illustrating the spread of change in fat fractions over the 12 months. There is wide variability of change in the semimembranosus muscle and to a lesser extent in the biceps femoris short head and semitendinosus muscles. This is possibly a result of the heavy infiltration at baseline and hence difficulties in ROI placements at follow up. While the biceps femoris short head muscle was not heavily fat infiltrated, it was difficult to accurately map the ROI between the initial and follow up scans, due to its shape. Figures 3 and 4 show fat fraction maps from two individual patients at baseline (a & c) and follow up (b & d). In Figure 3, the most notable change over time occurred in the medial gastrocnemius muscle (fat fraction increased by 21%), with smaller changes in the lateral gastrocnemius (4.6% increase), peroneus longus (8.1%) and soleus (1.7%). The fat fractions in the vastus lateralis and medialis also increased in this patient (7.0% and 7.2% increase in fat fraction, respectively), with more modest increases in the semimembranosus (4.7%), semitendinosus (4.4%), sartorius (2.6%) and gracilis (2.3%). In Figure 4, there are marked increases in the lateral and medial gastrocnemius (6.8% and 2.3% increase respectively), and in the thigh, increases in sartorius, gracilis and semitendinosus (4.5%, 4.5% and 4.7% respectively), with smaller increases in the rectus femoris (1.5%) and vastus lateralis (1.3%).

### Standardized Physical Testing

Over the 12 months period no significant changes were detectable in the patients’ standardized physical test measurements between baseline and follow-up (Table 4), despite evidence of change in some individuals relating to timed tests and myometry measurements, and a downward trend in all median measurements except ankle dorsiflexion.

**Table 4:** Minimum, maximum and median values of myometry, timed tests and FVC at baseline and follow-up in 32 paired individuals.

Muscle	Baseline min	Baseline max	Baseline median	12 months min	12 months max	12 months median	Difference (sig)
**Hip Flex (pounds)**	2.2	81.1	16.1	3.0	64.0	13.8	0.36
**Hip Abd (pounds)**	1.4	86.1	18.2	5.2	78.5	13.0	0.45
**Hip Add (pounds)**	1.6	58.9	14.1	4.0	74.8	9.1	0.11
**Knee Flex (pounds)**	1.9	66.1	18.6	0.0	70.5	17.7	0.10
**Knee Ext (pounds)**	4.3	156.6	26.3	4.4	142.3	20.5	0.82
**Ankle DF (pounds)**	5.6	86.3	38.0	4.0	93.6	46.0	0.16
**Stair climb time (secs)**	1.4	46.2	5.4	1.7	∞	6.4	0.08
**Stair descend time (secs)**	1.5	39.9	3.5	1.25	∞	4.5	0.24
**Chair rise time (secs)**	0.4	27.6	2.6	0.6	∞	3.3	0.55
**TUG (secs)**	4.3	50.5	12.0	4.1	∞	13.8	0.96
**10 metre time (secs)**	2.3	21.5	8.4	2.5	25.0	8.8	0.15
**6MWD (metres)**	67	625	312	50	718	353	0.77
**FVC (sit) (% predicted)** *	51	107	81	48	100	76	**0.001**
**FVC (lie) (% predicted)** *	36	105	71	28	100	66	**0.02**

Non-parametric paired Wilcoxon signed rank test was used. There was no significant difference found between the results at baseline and at follow up, apart from FVC.

∞ - the maximum time used for the analysis was infinity, to denote patients who were no longer able to do this test. Abd = abduction, Add = adduction, Flex = flexion, Ext = extension, DF = dorsiflexion, TUG = timed up and Go, 6MWD = six minute walk distance, FVC = forced vital capacity.

* FVC data from 25 patients.

The timed tests for some individuals suggested an increase in time to perform the stair climb, stair descend, 10 m walk, chair rise, and TUG test. However, again the increase was not significant for the group. The maximum value for the stair climb, stair descend, chair rise and TUG used was infinity (∞) as some of the patients were unable to perform this activity at follow up. The median six minute walk distance appeared to even show an improvement at follow up by 41 metres, but the change was not significant in the group. There were no significant correlations between the changes in the timed tests across 12 months and the changes in the muscle fat fractions measured by MRI.

The only statistically significant functional change over 12 months detected in our patient cohort was in forced vital capacity (FVC). The FVC measurements were obtained from 25 patients at baseline and follow up. The decrease in the FVC was significant both when sitting (median 81% predicted to 76% predicted at 12 months, p = 0.001) and when lying (median 71% predicted to 66% predicted, p = 0.02, Table 4).

## Discussion

This is the largest reported MRI study (n = 32) of patients with LGMD2I due to the common mutation in *FKRP*, and the first longitudinal natural history study to use quantitative MRI in LGMD2I. Quantitative fat imaging by the Dixon method demonstrated significant increases in fat replacement in 9 muscles across a 12 month period, while conventional physical testing of the patients did not demonstrate significant differences: indeed the median six minute walk distance appeared to increase, though not significantly. Qualitative grading of T1-weighted MRI images by a six-point scale was also shown to be insensitive. This study demonstrates the power of quantitative MRI to provide reliable objective outcome measures in a slowly progressing disease, as would be required in a clinical trial of therapy. It also provides new information about the rate of natural progression of pathology in individual muscle groups.

In Table 3 we have highlighted potential target muscles for the longitudinal assessment of pathological changes that were identified by our study. Targeting specific muscles for analysis in a longitudinal trial would maximize the power of a large therapeutic study. Muscles suitable for longitudinal analysis in LGMD2I were the medial gastrocnemius muscle in the lower leg and the vastus lateralis, gracilis and rectus femoris muscle in the thigh. These were identified not only because of fat fraction progression over 12 months, but because they demonstrated a wide range of fat fractions in patients at both the early and advanced stage of the disease and the ROIs were easy to delineate on follow-up. Unsuitable muscles included the tibialis anterior, which was only mildly affected at baseline and did not progress, and the biceps femoris long head which was already heavily fat-infiltrated at baseline and therefore unlikely to respond to therapy. Consistently well-positioned patients were essential for the delineation of muscles and the ROI placements at follow-up to enable reliable and uniform analysis of muscle pathology.

This work demonstrated that qualitative scoring of T_1_w images was not discriminating enough to detect progression of muscle pathology in patients with LGMD2I over a 12-month period, compared to the quantitative fat imaging method, which demonstrated significant increases in 9 out of the 14 muscles analyzed. The qualitative scoring is subjective and there are difficulties in assigning grades to some muscles due to the imprecise boundaries of the scoring system, especially between grades 2a, 2b and 3, leading to potential inconsistencies in scoring the T_1_w images. The interobserver repeatability of the T_1_w scores showed that whilst there was overall agreement in the scoring of the qualitative grades (65% of assessments), there was only 39% agreement in scoring grade 2b. The quantitative analysis of the images demonstrated good interobserver repeatability (inter-observer coefficient 1.43%) and was an objective measure of fat content in the individual muscles.

As shown in Table 4, the functional testing and physical strength measurements did not show any significant difference from baseline to follow up, apart from the FVC measurements: while these measurements are clinically important, their relationship to lower limb pathology is known to be indirect. The finding of decreased FVC can have diagnostic value in distinguishing LGMD2I patients from other LGMDs with normal respiratory function: it is important that lung function is monitored in any longitudinal trial, since respiratory care (including non-invasive ventilation) is an important treatment approach for LGMD2I patients. There were no significant correlations between the change in FVC and the change in fat fraction of any muscle group. Therefore, whilst functional assessments and strength tests are important as they provide clinically relevant information on the level of ability in these patients, objectively there was no statistical difference detected over 12 months in the tests. The median six minute walk distance, often used as the primary end-point of clinical trials of therapy, actually appeared to increase after 12 months demonstrating its unsuitability for use in slow progressing diseases. Learning effects from test repetition may affect these types of measurement.

Future work could include both asymptomatic and paediatric patients with the common *FKRP* mutation, in order to further define the natural history of the disease. Limitations of the study included the analysis of the quantitative Dixon scans at only one level. Ideally, one would like to assess the ROIs at all levels of potential target muscles and analyze the variability within muscles. This may give further information regarding the process of muscle damage occurring in this condition. In addition, while the technique can assess the changes in fat and water composition of the tissue, it cannot measure macromolecular species which may not be MR-visible. This means that other pathological processes which occur in LGMD2I, such as the development of fibrosis, cannot be assessed by this technique.

This study has shown that quantitative MR imaging by the Dixon technique provides an objective measurement of the muscle fat fraction that detects disease progression that cannot be identified by conventional functional testing, including measures such as the 6MWD presently used as primary trial end-points. In a slowly progressing neuromuscular condition, such as LGMD2I, quantitative MRI is able to demonstrate significant increases in muscle pathology in 9/14 of the muscles studied. Quantitative MRI could in the future be considered as a primary end-point of choice in the longitudinal monitoring of these patients in clinical trials.

## Supporting Information

File S1 (DOCX)
