# Supplementary material for: Quantitative Muscle MRI as an Assessment Tool for Monitoring Disease Progression in LGMD2I: A Multicentre Longitudinal Study
Source: PLoS One. 2013 Aug 14;8(8):e70993. doi: 10.1371/journal.pone.0070993 (PMC3743890; doi:10.1371/journal.pone.0070993)
Supplement: File S1 — (DOCX) [file pone.0070993.s001.docx]

**Supporting information**

**Details of loss to follow-up at 12 months**

Of the 38 patients recruited for the baseline examination, 32 were successfully followed up at 12 months. Loss of follow up was for the following reasons: two patients were pregnant at the time of follow-up (a contraindication to imaging for research in our ethical approval), one patient was undergoing treatment for a recently diagnosed malignancy and one patient opted to withdraw from the study. Two patients who did complete MRI assessment at 12 months were not included in the analyses due to scan re-positioning errors.

**Method and benefits of quantitative fat imaging by the Dixon method**

Traditionally, most neuromuscular research using MRI has used standard T_1_-weighted imaging to discern the presence or absence of muscular fat replacement, and to visually grade it on a subjective scale. Such sequences are fast and readily available on all scanner platforms. Due to muscle fat having a shorter T_1_ relaxation time than water, the fat signals are brighter. However, it is difficult to extract truly quantitative information from the signal intensity of these images, due to inhomogeneities in the applied B_1_ field, which mean that signal intensity is not proportional to fat fraction (Figure S1).

This inhomogeneity problem can be overcome by quantitative fat imaging using one of the class of Dixon methods. These methods acquire proton density weighted gradient echo images with 2,3, or more different echo times. Since the resonant frequencies of fat and water differ, the images at different echo times contain different combinations of fat and water signals. Mathematically we can model these and then calculate the components of the image that are fat and water respectively: we can also calculate the B_0_ inhomogeneity across the subject and correct for this [S1,S2]. At this point we have fat and water images which still contain B_1_ inhomogeneities (Figure S2). However, with proton density weighting the same inhomogeneities are present in the fat and water images, if we calculate a fat-fraction image then the B_1_ inhomogeneity will cancel out.

This is useful for cross-sectional and longitudinal studies as we now have a fat fraction image with every pixel containing the percentage fat in the MR signal (0-100%). As we have eliminated the B_0_ and B_1_ inhomogeneities, these results can be compared (i) between individuals and between scanning centers and (ii) longitudinally for the same individual. It is important that the leg is immobilised during the acquisition as movement will degrade the results: for skeletal muscle in the leg this is easy to arrange through strapping. As outlined in the Methods section, due to the need to use different scanners, two variations in the Dixon method were used [S1,S2]. We ensured that the implementations did not give different quantitative results by (i) agreeing equivalent proton-density weighted protocols of defined resolution, (ii) running quantitative acquisitions on phantoms of known water-fat composition on each implementation for comparison (by taking progressive slices through a water-fat boundary) and (iii) scanning control subjects to ensure that equivalent regions of interest could be marked on the images across the four centers.

**Repeatability of the Dixon method**

As the Dixon methods eliminate B_0_ and B_1_ inhomogeneities they are highly repeatable. The intrinsic repeatability of the technique and repositioning was studied in 6 control subjects by acquiring quantitative fat images of both the thigh and lower leg twice in close succession, removing the subjects from the scanner and repositioning them as per the protocol. The same ROIs analysed in the patients were analysed by one observer (KGH) and a Bland-Altman analysis was performed. The inter-scan repeatability coefficient in the fat fraction was 0.6% (with insignificant bias: 0.02%).

**Availability of the Dixon methods to clinicians**

Until very recently, quantitative Dixon methods were restricted to research centres and were not available as part of clinical scanner packages. This is starting to change, with major scanner manufacturers starting to offer sequences and analysis packages with new scanner platforms, indicating that the ease of access to these methods for clinical centers will steadily improve and the methods used in this research will become widely available.

**Figure S1 :** B_1_ inhomogeneity in conventional T_1_ weighted imaging


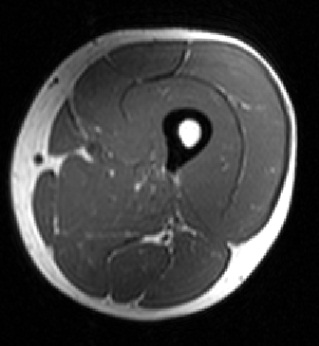


In standard T_1_ weighted imaging, the B_1_ (rf field) inhomogeneity that is always present means that the signal intensity of identical structures varies (such as that shown here in the subcutaneous fat, marked by arrows), so it is difficult to quantify muscle fat content. Usually the appearance is graded on a descriptive 6 point scale.

**Figure S2 :** Analysis workflow for quantitative Dixon MRI and removal of B_1_ inhomogeneity


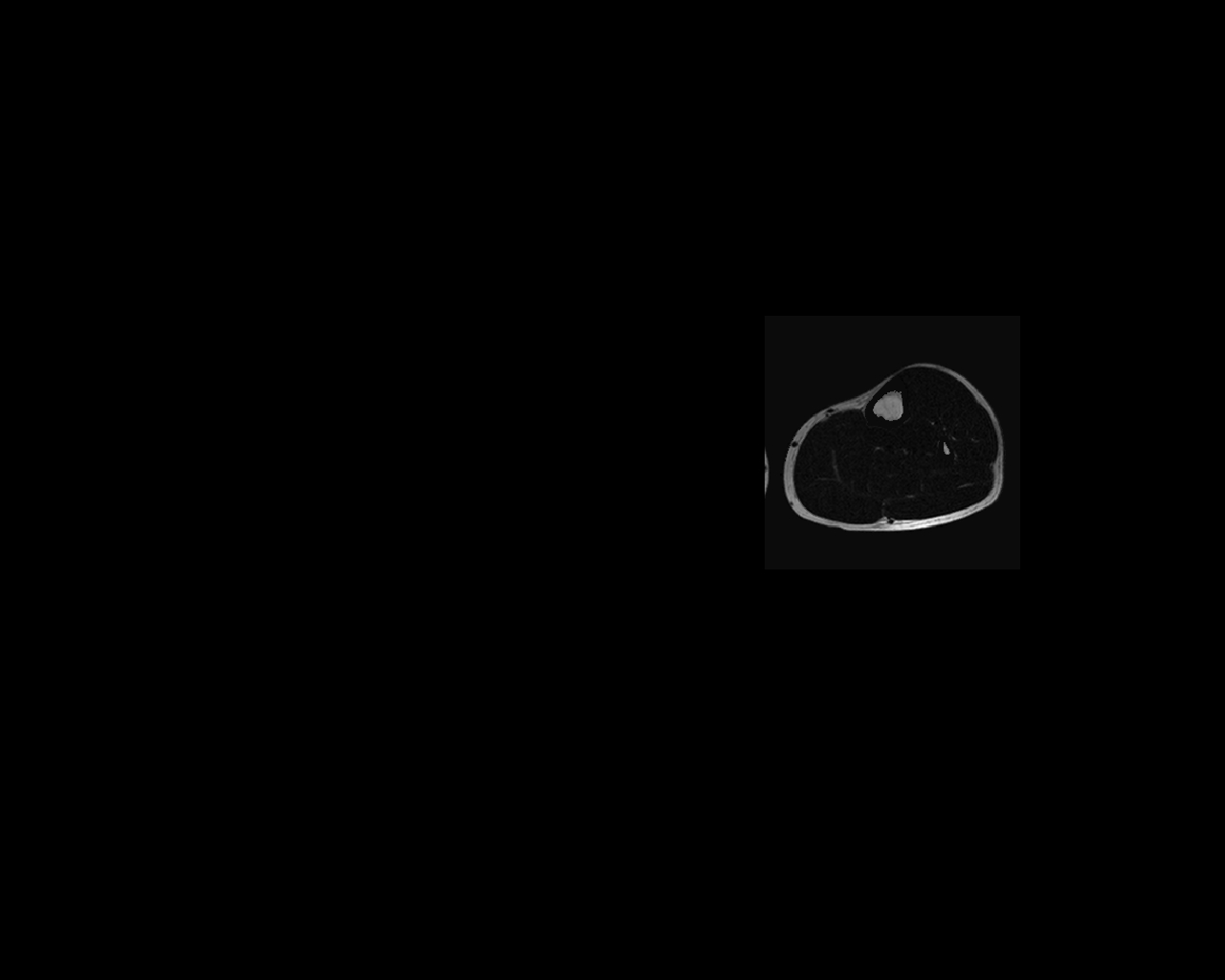

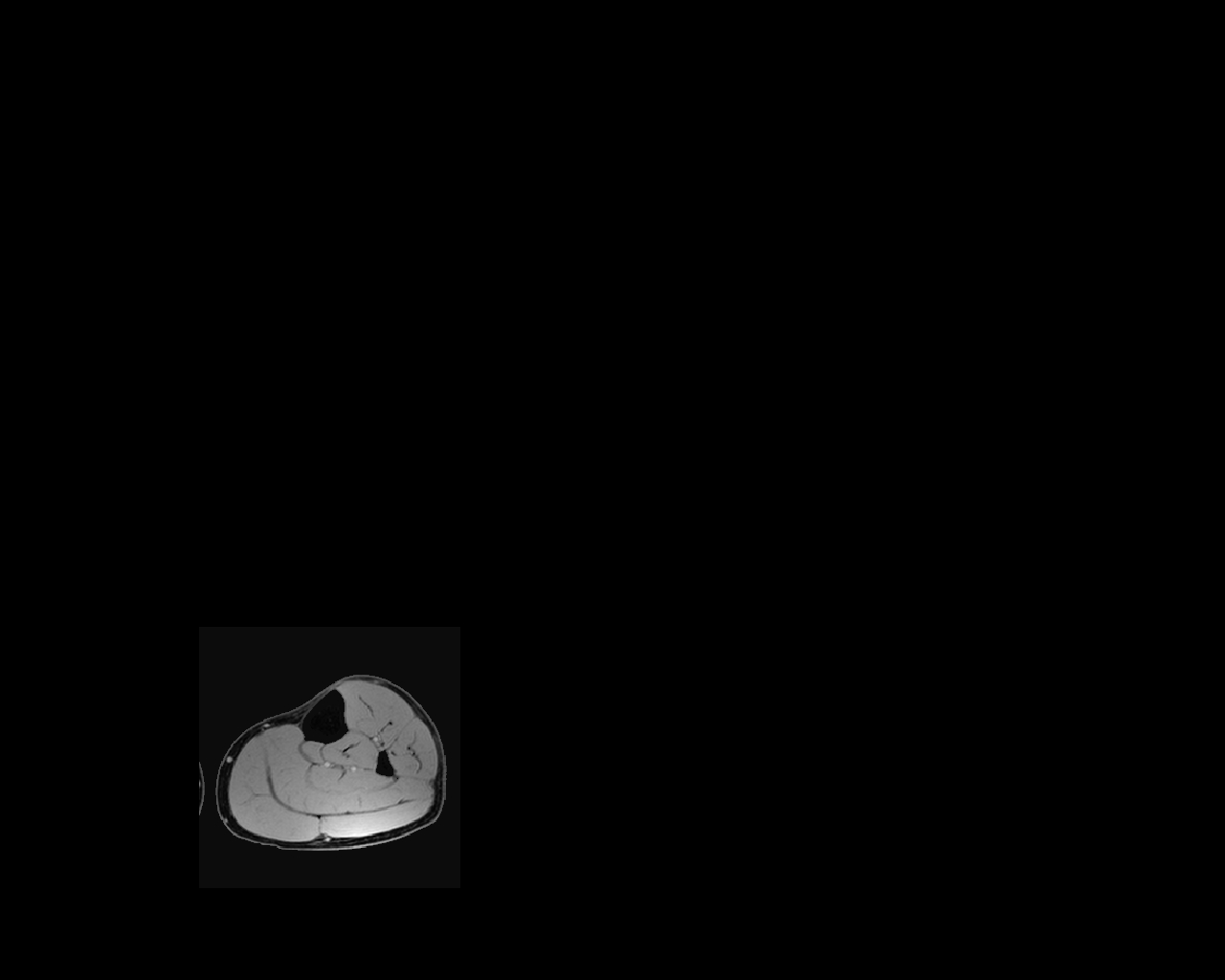

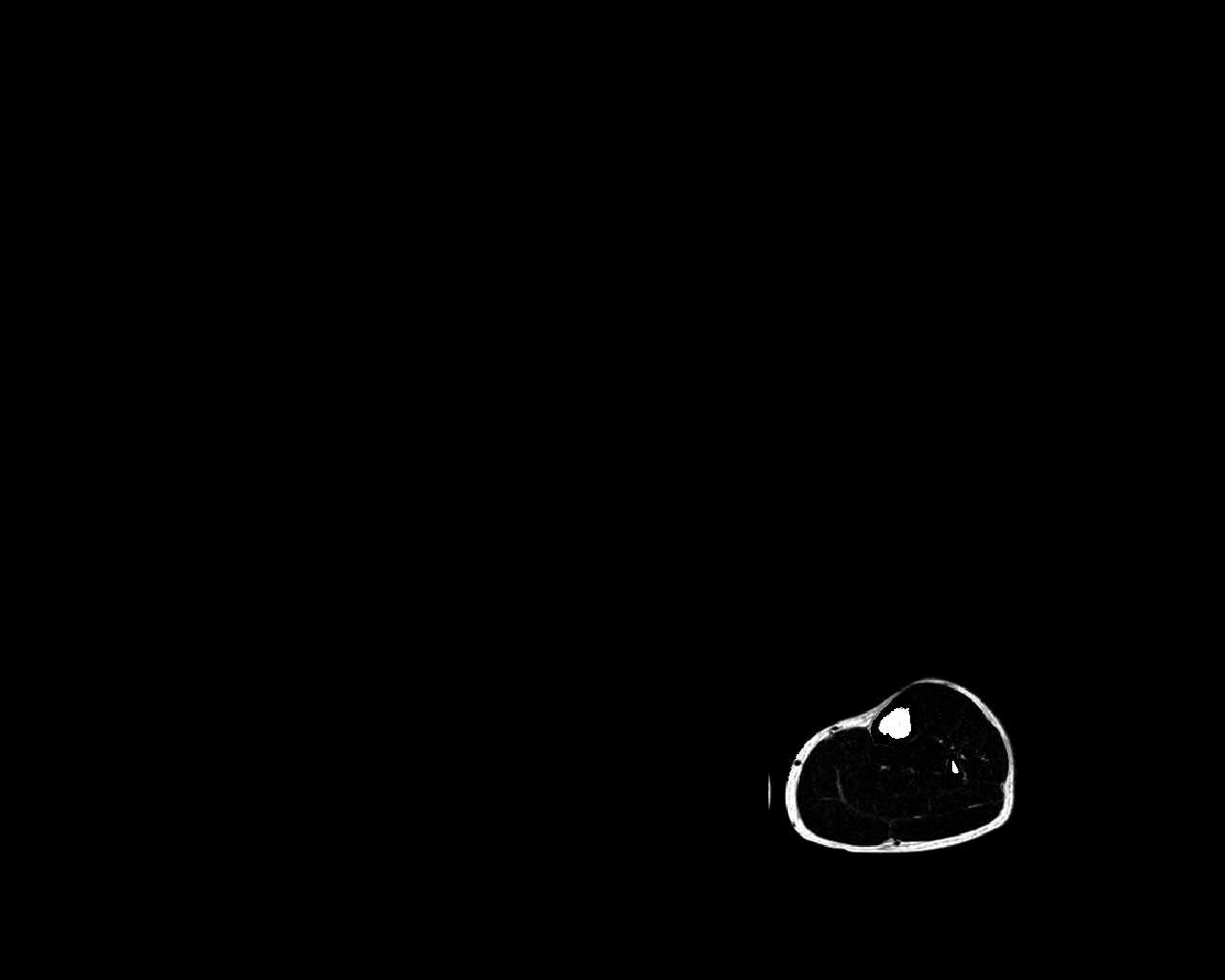

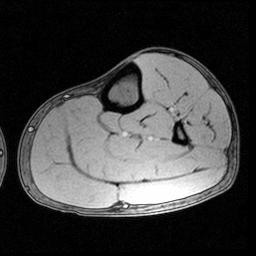

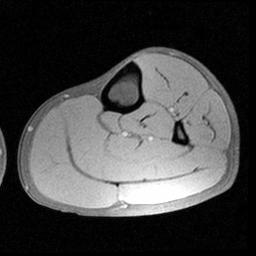

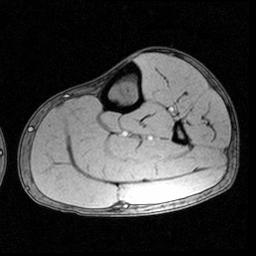


In the quantitative Dixon methods, 2 or 3 gradient echoes are acquired with different echo times which modulates the signals from fat and water, from left TE = 3.45,4.60,5.75ms. Note that in the first and third echoes fat and water signal cancels at the muscle/fat boundaries as the fat/water contributions are approx. 180^o^ out of phase, whereas it is additive in the second echo (in phase). These magnitude images still contain B_0_ and B_1_ inhomogeneity (brighter area marked by arrow). Phase data not shown.

The raw magnitude and phase data is analysed pixelwise^1,2^ to separate the water (*left*) and fat (*right*) contributions. B_0_ inhomogeneity has now been calculated and eliminated but the B_1_ inhomogeneity remains in these images (brighter area marked by arrow).

Since the B_1_ inhomogeneity is the same in both images, if we calculate the fat fraction (intensity in fat image over total intensity of fat+water), then the inhomogeneity cancels out and uniform structures have a uniform signal intensity from 0%-100% fat in the final image, which can then be measured by region-of-interest analysis. Some scanner implementations derive the same corrections from 2 echoes, as outlined in Reference S1.

| **Table S1:** Percentage of LGMD2I patients in each category of semi-quantitative grading for individual muscles at 12 month follow up using the qualitative grading scale, with the median grade for each muscle. | | | | | | | |
| --- | --- | --- | --- | --- | --- | --- | --- |
| **Semi quantitative grade**  **Muscle** | **0** | **1** | **2a** | **2b** | **3** | **4** | **Median grade** |
| Gluteus Maximus (GM) | 0 | 3.1 | 12.5 | 3.1 | 25.0 | 56.3 | 4 |
| Biceps Femoris long head (BFLH) | 3.1 | 3.1 | 9.4 | 9.4 | 9.4 | 65.6 | 4 |
| Semitendinosus (ST) | 3.1 | 6.3 | 9.42 | 9.4 | 18.8 | 53.1 | 4 |
| Semimembranosus (SM) | 3.1 | 6.3 | 12.5 | 15.6 | 18.8 | 43.8 | 3 |
| Biceps Femoris short head (BFSH) | 3.1 | 9.4 | 21.9 | 25.0 | 21.9 | 18.8 | 2b |
| Sartorius (SAR) | 3.1 | 6.3 | 37.5 | 18.8 | 21.9 | 12.5 | 2b |
| Vastus Medialis (VM) | 9.4 | 12.5 | 18.8 | 6.3 | 28.1 | 25.0 | 3 |
| Gracilis (GRAC) | 3.1 | 12.5 | 34.4 | 12.5 | 25.0 | 12.5 | 2b |
| Vastus Lateralis (VL) | 6.3 | 12.5 | 9.4 | 25.0 | 28.1 | 18.8 | 2b |
| Rectus Femoris (RF) | 9.4 | 15.6 | 21.9 | 21.9 | 15.6 | 15.6 | 2b |
| Medial Gastrocnemius (MG) | 6.3 | 6.3 | 12.5 | 28.1 | 18.8 | 28.1 | 2b |
| Lateral Gastrocnemius (LG) | 6.3 | 12.5 | 15.6 | 18.8 | 37.5 | 9.4 | 2b |
| Peroneus Longus (PL) | 6.3 | 6.3 | 40.6 | 25.0 | 15.6 | 6.3 | 2a |
| Soleus (SOL) | 3.1 | 12.5 | 28.1 | 28.1 | 21.9 | 6.3 | 2b |
| Tibialis Anterior (TA) | 9.4 | 25.0 | 56.3 | 9.4 | 0 | 0 | 2a |

**References:**

S1. Coombs BD, Szumowski J, Coshow W. Two-point Dixon technique for water-fat signal decomposition with B_0_ inhomogeneity correction. Magn Reson Med 1997; 38(6):884-889.

S2. Glover GH, Schneider E. Three-point Dixon technique for true water/fat decomposition with *B_0_* inhomogeneity correction. Magn Reson Med 1991; 18(2):371-383.
